# Kinetics of pro- and anti-inflammatory spike-specific cellular immune responses in long-term care facility residents after COVID-19 mRNA primary and booster vaccination: a prospective longitudinal study in Japan

**DOI:** 10.1186/s12979-024-00444-1

**Published:** 2024-06-22

**Authors:** Tomoyuki Kakugawa, Yusuke Mimura, Yuka Mimura-Kimura, Keiko Doi, Yuichi Ohteru, Hiroyuki Kakugawa, Keiji Oishi, Masahiro Kakugawa, Tsunahiko Hirano, Kazuto Matsunaga

**Affiliations:** 1 Department of Pulmonology and Gerontology, Graduate School of Medicine, Yamaguchi University, Ube, Japan; 2 Medical Corporation WADOKAI, Hofu Rehabilitation Hospital, Hofu, Japan; 3 Department of Respiratory Medicine, National Hospital Organization Yamaguchi Ube Medical Center, Ube, Japan; 4 The Department of Clinical Research, National Hospital Organization Yamaguchi Ube Medical Center, Ube, Japan; 5 Department of Respiratory Medicine and Infectious Disease, Graduate School of Medicine, Yamaguchi University, Ube, Japan

**Keywords:** COVID-19, Older adults, Frailty, Vaccination, SARS-CoV-2, Cell-mediated immunity, Interleukin, IFN-γ, IL-10

## Abstract

**Background:**

The magnitude and durability of cell-mediated immunity in older and severely frail individuals following coronavirus disease 2019 (COVID-19) vaccination remain unclear. A controlled immune response could be the key to preventing severe COVID-19; however, it is uncertain whether vaccination induces an anti-inflammatory cellular immune response. To address these issues, a 48-week-long prospective longitudinal study was conducted. A total of 106 infection-naive participants (57 long-term care facility [LTCF] residents [median age; 89.0 years], 28 outpatients [median age; 72.0 years], and 21 healthcare workers [median age; 51.0 years]) provided peripheral blood mononuclear cell (PBMC) samples for the assessment of spike-specific PBMC responses before primary vaccination, 24 weeks after primary vaccination, and three months after booster vaccination. Cellular immune responses to severe acute respiratory syndrome coronavirus 2 spike protein were examined by measuring interferon (IFN)-γ, tumor necrosis factor (TNF), interleukin (IL)-2, IL-4, IL-6, and IL-10 levels secreted from the spike protein peptide-stimulated PBMCs of participants.

**Results:**

LTCF residents exhibited significantly lower IFN-γ, TNF, IL-2, and IL-6 levels than healthcare workers after the primary vaccination. Booster vaccination increased IL-2 and IL-6 levels in LTCF residents comparable to those in healthcare workers, whereas IFN-γ and TNF levels in LTCF residents remained significantly lower than those in healthcare workers. IL-10 levels were not significantly different from the initial values after primary vaccination but increased significantly after booster vaccination in all subgroups. Multivariate analysis showed that age was negatively associated with IFN-γ, TNF, IL-2, and IL-6 levels but not with IL-10 levels. The levels of pro-inflammatory cytokines, including IFN-γ, TNF, IL-2, and IL-6, were positively correlated with humoral immune responses, whereas IL-10 levels were not.

**Conclusions:**

Older and severely frail individuals may exhibit diminished spike-specific PBMC responses following COVID-19 vaccination compared to the general population. A single booster vaccination may not adequately enhance cell-mediated immunity in older and severely frail individuals to a level comparable to that in the general population. Furthermore, booster vaccination may induce not only a pro-inflammatory cellular immune response but also an anti-inflammatory cellular immune response, potentially mitigating detrimental hyperinflammation.

**Supplementary Information:**

The online version contains supplementary material available at 10.1186/s12979-024-00444-1.

## Background

The spread of severe acute respiratory syndrome coronavirus 2 (SARS-CoV-2) caused a global outbreak of coronavirus disease 2019 (COVID-19), leading to a significant number of fatalities. As vaccination rates increase and the Omicron variant replaces earlier strains, a considerable decrease in COVID-19 morbidity and mortality has been observed [1]. However, older adults and individuals with multiple comorbidities have higher COVID-19 mortality and morbidity rates than those with influenza [2, 3]. Among these individuals, long-term care facility (LTCF) residents are particularly vulnerable to COVID-19 outbreaks, given the high infectivity of SARS-CoV-2.

The COVID-19 vaccine remains crucial in safeguarding older and severely frail populations against severe COVID-19. However, recent evidence suggests that antibody levels following COVID-19 vaccination may decline more rapidly in older adults than in younger or middle-aged individuals [4–13]. We previously reported that following COVID-19 mRNA primary vaccination, LTCF residents displayed lower neutralizing antibody activity against the wild-type and Delta variants of SARS-CoV-2 than the general population [13]. Although T-cells may maintain their defense capabilities against severe disease [14–21], the magnitude and durability of cell-mediated immunity in older and severely frail individuals, such as LTCF residents, compared to those in the general population remain unclear.

Advanced age is the most significant risk factor for severe COVID-19 [22–25]. Nevertheless, even among older adults, the clinical outcomes of SARS-CoV-2 infection exhibit considerable variability. While some individuals experience only mild symptoms or remain asymptomatic, others develop severe illness [26–30]. This diversity in immune responses was particularly pronounced before the availability of vaccines [26–30]. With the progression of vaccination, the severity of COVID-19 in older adults has significantly decreased [1], but the diversity in immune responses continues to be observed [25]. This diversity may be attributed to various factors; however, the specific underlying mechanisms remain to be elucidated.

Markedly elevated levels of pro-inflammatory cytokines (including interleukin [IL]-6) are associated with critical and fatal COVID-19, and blocking the inflammatory pathway may prevent disease progression [31, 32]. The cytokine storm observed during the initial stages of SARS-CoV-2 infection can precipitate severe disease when the deleterious effects of the immune response outweigh the immediate antiviral benefits [33]. High cytokine and chemokine levels during this phase are associated with an increased likelihood of experiencing a severe form of the disease and a higher risk of mortality [31, 34]. Interventions targeting the virus demonstrate effectiveness in the early stages, whereas those targeting the immune response show greater efficacy during later stages. Data from randomized trials support the use of glucocorticoids and tocilizumab for severe cases of COVID-19 [35–39], indicating the significance of harmful hyperinflammatory responses during the advanced stages of the disease.

Considering the points mentioned above, eliciting an immune response that controls excessive immune responses may be crucial for preventing the development of severe COVID-19; however, researchers are unsure whether vaccination induces such a response. Moreover, it is unclear whether these responses are associated with factors such as age, sex, nutritional status, or underlying comorbidities.

To address these issues, we designed a one-year prospective longitudinal study focusing on pro- and anti-inflammatory spike-specific cellular immune responses of peripheral blood mononuclear cell (PBMC) in older and severely frail individuals within the distinctive context of COVID-19 vaccination, wherein individuals were uniformly exposed to the same antigenic stimulus. We examined responses following both primary and booster vaccinations, including not only LTCF residents but also outpatients and healthcare workers. This comprehensive approach enabled comparisons among older adults requiring extended care, those living independently at home, and healthy younger individuals.

Our results could be useful for the development of robust booster strategies as protective measures against the development of severe COVID-19 in older and severely frail individuals, such as LTCF residents.

## Methods

### Study design and population

Written informed consent was obtained from all participants or their legal guardians. The study protocol adhered to the Declaration of Helsinki and was approved by the Institutional Review Board of Yamaguchi University Hospital (registration no. 2020-214). This prospective longitudinal study was registered in the UMIN Clinical Trials Registry (UMIN Trial ID: UMIN000043558). The detailed protocol for this study is available at https://center6.umin.ac.jp/cgi-open-bin/ctr/ctr_view.cgi?recptno=R000049712. The objectives of this prospective study were to: 1) evaluate humoral immune responses after COVID-19 vaccination, 2) evaluate cellular immune responses after COVID-19 vaccination, and 3) investigate the relationship between intestinal microbiomes and the immunogenicity and durability of the COVID-19 vaccine. We have previously published an interim report on the humoral immune responses post COVID-19 vaccination [13]. The participants in this report on cellular immune responses are the same as those in the interim report [13]. However, the schedule for blood sampling in this study on cellular immune responses was different from that in our previous report on humoral immune responses.

The design and population of this prospective longitudinal study were previously described [13] but are briefly reiterated here for clarity. This study was conducted from March 5, 2021, to July 6, 2022, and included LTCF residents, outpatients, and healthcare workers who had not yet received their first COVID-19 vaccine dose. The LTCFs included four nursing homes and one long-term care hospital in Yamaguchi, Japan, and outpatient clinics included Yamaguchi University Hospital or Hofu Rehabilitation Hospital in Yamaguchi, Japan. All participants were tested for antibodies that target the viral nucleocapsid protein [IgG(N)] to rule out COVID-19 breakthrough infection during the study period (at baseline and 8, 12, 24, and 48 weeks after the first dose). A nucleic acid amplification test for SARS-CoV-2 was performed if any COVID-19-associated symptom (eg. rhinorrhea and/or nasal congestion, headache, sore throat, cough, chills/rigors, fever, myalgias, confusion, anosmia or other smell abnormalities, chest pain or pressure, nausea/vomiting, diarrhea, fatigue, dyspnea, taste abnormalities) or exposure to a SARS-CoV-2-infected person was reported. The eligibility criteria included the absence of SARS-CoV-2 infection, and individuals with positive results of IgG(N) or nucleic acid amplification test for SARS-CoV-2 were excluded from the final analyses. All participants were asked to provide peripheral blood samples for the assessment of spike-specific PBMC responses before and 24 and 48 weeks after primary vaccination with the BNT162b2 (Pfizer-BioNTech) COVID-19 vaccine (two intramuscular doses of 30 mcg each were given three weeks apart). Participants who had undergone at least two assessments of the spike-specific PBMC response from the baseline period were included in the final analysis. The endpoint of the study for any participant was defined as 350 days after administration of the first vaccine dose, death, or lack of follow-up.

Frailty and nutritional parameters were recorded at baseline. Frailty parameters included Eastern Cooperative Oncology Group Performance Status (ECOG-PS) [0: Fully active; no performance restrictions. 1: Strenuous physical activity restricted; fully ambulatory and able to carry out light work. 2: Capable of all self-care but unable to carry out any work activities. Up and about >50% of waking hours. 3: Capable of only limited self-care; confined to bed or chair >50% of waking hours. 4: Completely disabled; cannot carry out any self-care; totally confined to bed or chair.] [40], functional independence measure (FIM) [a proprietary instrument that assesses patient disability in 13 aspects of motor function and five aspects of cognitive function] [41], and Mini-Mental State Examination (MMSE) [scored on a 30-point scale, with items that assess orientation (temporal and spatial; 10 points), memory (registration and recall; 6 points), attention/concentration (5 points), language (verbal and written; 8 points), and visuospatial function (1 point)] [42]. Nutritional parameters included body mass index, serum total protein level, and serum albumin level. Information on comorbidities and laboratory data (as shown in Additional File 1) were also obtained.

### Isolation of PBMCs

PBMCs were isolated from 16 mL of whole blood in BD Vacutainer CPT tubes (BD Biosciences, Franklin Lakes, NJ, USA) or SepMate-50 tubes (STEMCELL Technologies, Vancouver, Canada) according to the manufacturer’s instruction. The isolation of PBMC was initiated within one hour from the time of blood collection. The PBMCs were frozen in Cellbanker 1plus (ZENOGEN PHARMA, Fukushima, Japan) at −80 ℃ overnight and stored in liquid nitrogen until further use.

### PBMC stimulation with synthetic peptides of SARS-CoV-2 spike protein

Cellular immune responses to SARS-CoV-2 spike protein were examined by measuring interferon (IFN)-γ, tumor necrosis factor (TNF), IL-2, IL-4, IL-6, and IL-10 secreted from the spike protein peptide-stimulated PBMCs of participants. Frozen PBMCs were thawed and incubated in RPMI 1640 medium (Merck, Rahway, NJ, USA) supplemented with 10% heat-inactivated fetal bovine serum (Gibco, Paisley, UK), 100 U/mL penicillin, 100 µg/mL streptomycin, and 2 mM L-glutamine overnight. The PBMCs were transferred to a 96-well V-bottom (Corning, Kennebunk, ME, USA) at 1 × 10^6^ /100 µl/well. A peptide library of 15-mers overlapping by 11 amino acids spanning the full-length spike protein sequence derived from the wild-type virus (Wuhan strain) (315 peptides in total, PreMix SARS-CoV-2 Spike Glycoprotein; JPT Peptide Technologies, Berlin, Germany) were contained in the culture medium at 2 µg/mL per peptide, and 100 µL of the peptide-containing medium was added to the PBMC culture (final concentration 1 µg/mL/peptide). After incubation for 18 h, the culture supernatants were harvested after centrifugation of the plate at 1500 rpm for 3 min and stored at −80 °C until use. IFN-γ, IL-2, TNF, IL-4, IL-6, and IL-10 levels in the supernatants were measured with a Human Th1/Th2 Cytokine Cytometric Beads Array Kit II (BD, San Diego, CA, USA) and FACSLyric (BD, San Diego, CA, USA) according to the manufacturer’s instructions. The results were analyzed using FCAP Array software ver. 3.0 (BD, San Diego, CA, USA). The lower limit of detection for each cytokine was established at twice the median fluorescence intensity of the negative control (0 pg/mL). The lower detection limits for each are as follows: IFN-γ 1.2 pg/mL; TNF, 1.8 pg/mL; IL-2, 2.9 pg/mL; IL-6, 1.6 pg/mL; IL-10, 1.2 pg/mL; IL-4, 1.3 pg/mL. Values below these thresholds were replaced with the detection limit for purposes of statistical analysis and graph plotting. In our analyses, we have classified the following cytokines as pro-inflammatory: IFN-γ, TNF, IL-2, and IL-6; the following is classified as anti-inflammatory: IL-10.

### Serological assays

In this study, we utilized our previously reported results of serological testing for antibodies against the receptor-binding domain (RBD) of the S1 subunit of the viral spike protein [IgG(S-RBD)], IgG(N), and surrogate virus neutralization test (sVNT; cPass SARS-CoV-2 Neutralization Antibody Detection Kit, Genscript Biotech Corporation, Piscataway, NJ, USA) [13] to investigate the correlation between the spike-specific PBMC responses tested in this study and the humoral immune responses demonstrated in our prior interim report [13]. The sVNT identifies functional antibodies that neutralize the interaction between the spike protein RBD and human angiotensin-converting enzyme 2 [43–45] for the wild-type virus as well as the Delta (B.1.617.2) and Omicron (B.1.1.529, sublineage BA.1) variants.

### Statistical analysis

The data were stratified into three groups: healthcare workers, outpatients, and LTCF residents. Values were summarized as median and interquartile range (IQR) for continuous variables and as frequencies (percentage) for categorical variables. Differences in cytokine levels between time points were tested pairwise using one-sample Wilcoxon test with Bonferroni correction for multiple comparisons, whereas between-group differences were tested using Fisher’s exact test for categorical variables and the Mann-Whitney test or the Kruskal–Wallis test for numerical variables. All pairwise comparisons after the Kruskal–Wallis test were performed using Dunn’s test with the Bonferroni correction for multiple testing. Correlations between variables were calculated using Spearman's rank correlation coefficient (rS). Factors causing variation in the IFN-γ, TNF, IL-2, IL-6, and IL-10 levels were analyzed using multiple regression analyses (MRAs) by setting each type of cytokine as an objective variable and the following demographic/clinical factors as explanatory variables: age, sex, number of comorbidities, immunosuppressive status, Eastern Cooperative Oncology Group Performance Status (ECOG-PS), serum albumin, estimated glomerular filtration rate (e-GFR), glycated hemoglobin, and booster vaccination type. The optimal regression model was constructed using repeated stepwise selection from the explanatory variables. Specifically, we performed three cycles of forward selection, allowing for the re-entry of previously deleted variables, using a P value threshold of 0.05. The overall model fitness was monitored at each step by computing the adjusted coefficient of determination. During the selection process, “age” was included in the model as a control variable to avoid confounding effects on other parameters. The practical significance of each parameter retained in the regression model was interpreted based on its standardized partial regression coefficient, which corresponds to the partial correlation coefficient (*r*_*p*_) and takes values between −1.0 and 1.0. In reference to Cohen's criterion for the effect size of the correlation coefficient [46], we regarded 0.20≦|*r*_*p*_|<0.3, 0.30≦|*r*_*p*_|<0.5, and 0.5≦|*r*_*p*_| as indicative of a “weak,” “moderate,” and “strong” correlations, respectively. All statistical analyses were performed using StatFlex for Windows Ver. 7 (Artech Inc., Osaka, Japan). Scatter and box-and-whisker plots were generated using JMP Pro 16.1.0 (SAS Institute Inc., Cary, NC, USA).

## Results

### Study population and serological assays

The final study sample comprised 106 infection-naive participants (57 LTCF residents, 28 outpatients, and 21 healthcare workers). The distribution of demographic characteristics and data on coexisting conditions among participants at baseline are shown in Additional File 1. The sample population consisted of 100% Asians, 58.5% of whom were females. The median age of the LTCF residents was 89.0 years, with an IQR of 83.0–93.0 years. Among LTCF residents, 57.9% had an ECOG-PS score of 4 and 29.8% had an ECOG-PS score of 3. The median FIM and MMSE of the LTCF residents were 32.0 and 7.5, respectively, with IQR of 21–67 for FIM and 0–16 for MMSE. The number of participants included in the final analysis who underwent assessment of spike-specific PBMC responses at each period is shown in Additional File 2. One participant refused to complete both vaccination doses and was thus excluded from the final analysis. The remaining participants completed two vaccination doses with the BNT162b2 (Pfizer-BioNTech) COVID-19 vaccine in the primary vaccine series. From 24 to 48 weeks after the primary vaccination, two participants failed to receive booster vaccinations and were subsequently excluded from the final analysis at 48 weeks. The remaining participants received booster vaccinations from 24 to 48 weeks after the primary vaccination. Therefore, the assessment at 48 weeks after the first dose took place approximately three months after the booster vaccination, wherein all healthcare workers, 14 of 26 outpatients, and 15 of 50 LTCF residents received the BNT162b2 (Pfizer-BioNTech) COVID-19 vaccine. Meanwhile, the remaining 12 of 26 outpatients and 35 of 50 LTCFs residents were administered the mRNA-1273 (Moderna) COVID-19 vaccine. No participants were identified as being infected with SARS-CoV-2 during the study period. However, five participants showed positive IgG(N) results during the study period; these participants were considered to have been infected asymptomatically with SARS-CoV-2 during the study period and excluded from the final analysis.

### Spike-specific PBMC response kinetics

**Fig. 1:**
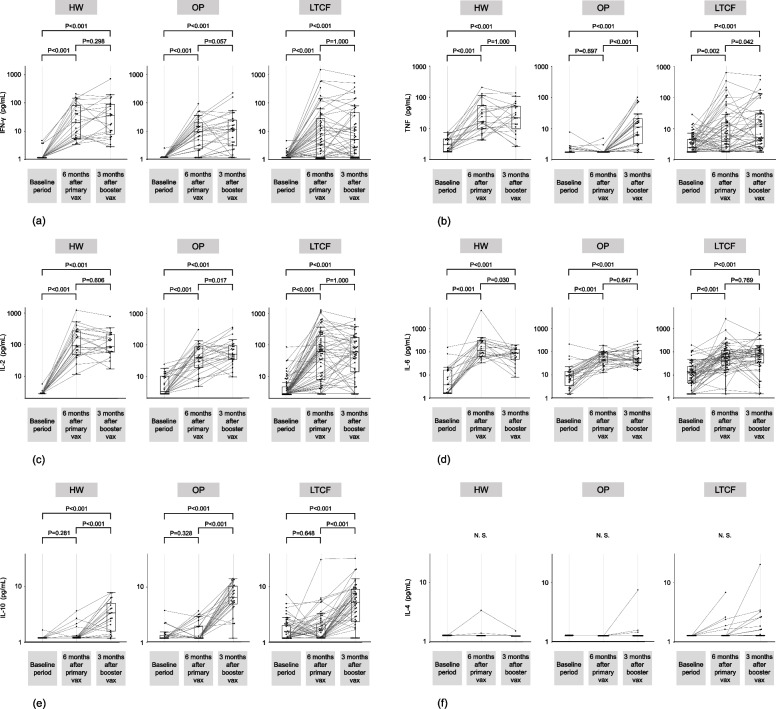
Kinetics of spike-specific peripheral blood mononuclear cell responses. Cellular immune responses to severe acute respiratory syndrome coronavirus 2 (SARS-CoV-2) spike protein, as examined by measuring (**a**) interferon (IFN)-γ, (**b**) tumor necrosis factor (TNF), (**c**) interleukin (IL)-2, (**d**) IL-6, (**e**) IL-10, and (**f**) IL-4 secreted from the spike protein peptide-stimulated peripheral blood mononuclear cells of participants before and six months after the primary vaccination and three months after the booster vaccination. Participants are stratified into three subgroups: healthcare workers (HW), outpatients (OP), and residents of long-term care facilities (LTCF). Each dot represents an individual participant, and the lines indicate corresponding pairs. The levels of each cytokine are logarithmically transformed. Boxes span the interquartile range; the line within each box denotes the median, and the whiskers are the largest and smallest values within the range of ±1.5-fold in the interquartile range from the first and third quartiles. *P* values were determined using one-sample Wilcoxon test with Bonferroni correction for multiple comparisons. N.S.; not significant

Figure 1 shows the kinetics of the spike-specific PBMC responses before and six months after the primary vaccination as well as three months after the booster (third dose) vaccination. IFN-γ, IL-2, and IL-6 levels were significantly elevated after the primary vaccination compared to baseline levels in all subgroups. TNF levels were also significantly elevated after the primary vaccination compared to baseline levels in healthcare workers and LTCF residents, but not in outpatients. IFN-γ levels were not significantly elevated after booster vaccination compared to levels before booster vaccination in any subgroup. IL-6 levels were not significantly elevated after booster vaccination compared to levels before booster vaccination in outpatients and LTCF residents. However, IL-6 levels were significantly decreased after booster vaccination compared to levels before booster vaccination in healthcare workers. TNF levels did not significantly increase after booster vaccination compared to levels before booster vaccination in healthcare workers but did increase in outpatients and LTCF residents. Similarly, IL-2 levels did not significantly increase after booster vaccination compared to levels before booster vaccination in healthcare workers or LTCF residents but did increase in outpatients. IL-10 levels were not significantly elevated after primary vaccination compared to those before primary vaccination in any subgroup but were significantly elevated after the booster vaccination compared to baseline and pre-booster vaccination levels in all subgroups. IL-4 levels were not significantly elevated following primary or booster vaccination in any subgroup.

### Comparison of the spike-specific PBMC response among subgroups

**Fig. 2:**
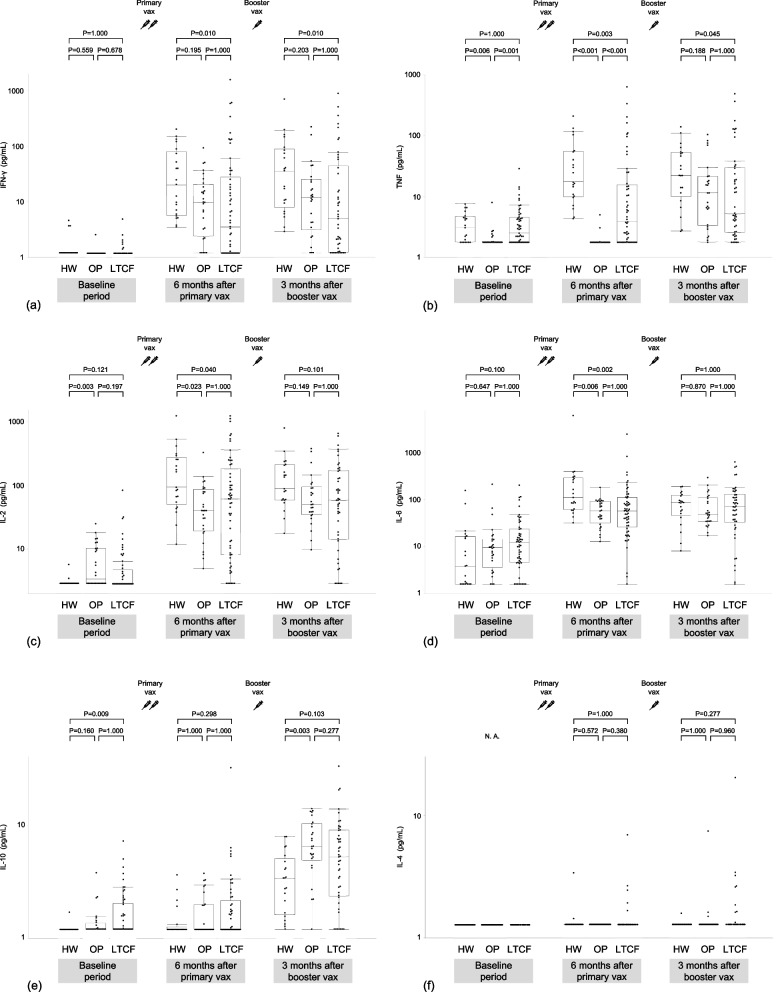
Comparison of the spike-specific peripheral blood mononuclear cell response among subgroups. Cellular immune responses to severe acute respiratory syndrome coronavirus 2 (SARS-CoV-2) spike protein, as examined by measuring (**a**) interferon (IFN)-γ, (**b**) tumor necrosis factor (TNF), (**c**) interleukin (IL)-2, (**d**) IL-6, (**e**) IL-10, and (**f**) IL-4 secreted from the spike protein peptide-stimulated peripheral blood mononuclear cells of participants before and six months after the primary vaccination, and three months after the booster vaccination. Participants are stratified into three subgroups: healthcare workers (HW), outpatients (OP), and residents of long-term care facilities (LTCF). Each dot represents the individual participant. The levels of each cytokine were logarithmically transformed into a plot. Boxes span the interquartile range; the line within each box denotes the median, and the whiskers are the largest and smallest values within the range of ±1.5-fold in the interquartile range from the first and third quartile. Between-group differences were tested pairwise using Dunn’s test for multiple comparisons. *P* values are indicated for each plot. N.A.; not applicable

Figure 2 shows a comparison of spike-specific PBMC responses among the subgroups. LTCF residents exhibited significantly lower IFN-γ, TNF, IL-2, and IL-6 levels than healthcare workers after the primary vaccination. Outpatients had significantly lower TNF, IL-2, and IL-6 levels than healthcare workers but comparable IFN-γ levels after the primary vaccination. After booster vaccination, IL-2 and IL-6 levels in LTCF residents and outpatients, as well as TNF levels in outpatients, were comparable to those in healthcare workers. In contrast, LTCF residents exhibited significantly lower IFN-γ and TNF levels than healthcare workers after the booster vaccination. No significant difference in IL-10 levels following primary vaccination was observed among the subgroups, whereas outpatients exhibited significantly higher IL-10 levels than healthcare workers after booster vaccination.

### Correlations between age and IFN-γ, TNF, IL-2, IL-6, and IL-10 levels

**Fig. 3:**
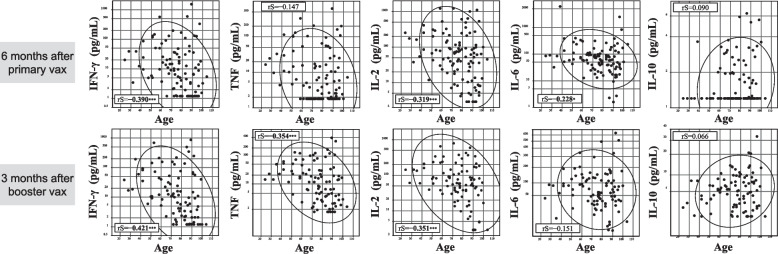
Correlations between age and IFN-γ, TNF, IL-2, IL-6, and IL-10 levels. The upper and lower rows show the correlations six months after primary vaccination and three months after booster vaccination, respectively. rS: Spearman rank correlation coefficient. Values of rS are shown in bold when P values are less than 0.05: * *P*<0.05, ** *P*<0.01, and *** *P*<0.001. The levels of each cytokine are logarithmically transformed. The 90% confidence ellipse region was drawn assuming a near-Gaussian distribution of the values

Figure 3 shows the correlation between age and IFN-γ, TNF, IL-2, IL-6, and IL-10 levels. Age exhibited negative correlations with IFN-γ, IL-2, and IL-6 levels six months after the primary vaccination (rS: −0.390, −0.319, and −0.228, respectively) and with IFN-γ, TNF, and IL-2 levels three months after the booster vaccination (rS: −0.421, −0.354, and −0.351, respectively). No significant correlation was observed between age and IL-10 level.

### MRA of possible factors responsible for the variation in IFN-γ, TNF, IL-2, IL-6, and IL-10 levels

Table 1 presents the MRA results. TNF levels six months after the primary vaccination were negatively associated with age and positively associated with the ECOG-PS score and serum albumin levels. IFN-γ levels three months post booster vaccination were also negatively associated with age and positively associated with the ECOG-PS score and serum albumin levels. IL-10 levels three months after the booster were negatively associated with the ECOG-PS score and serum albumin levels. IL-2 levels showed a weak positive association with e-GFR three months after the booster.

**Table 1:** Multiple regression analyses of possible factors underlying IFN-γ, TNF, IL-2, IL-6, and IL-10 level variations

			Univariate analysis	Multivariate analysis of factors responsible for variation in cytokine levels									
		*n*	Age	R	Age	Sex^a^	No. of Com	Immuno-suppression^b^	ECOG-PS	Serum albumin	e-GFR	HbA1c	Booster vaccination type, mRNA-1273 (Moderna)^c^
6 months after primary vaccination	IFN-γ	106	-0.321***	0.321	-0.321***	----	----	----	----	----	----	----	----
	TNF	106	-0.205	0.391	-0.332*	----	----	----	0.527***	0.318*	----	----	----
	IL-2	106	-0.320***	0.320	-0.320***	----	----	----	----	----	----	----	----
	IL-6	106	-0.235*	0.235	-0.235*	----	----	----	----	----	----	----	----
	IL-10	106	0.163	0.120	0.163	----	----	----	----	----	----	----	----
3 months after booster vaccination	IFN-γ	93	-0.386***	0.476	-0.317*	----	----	----	0.371*	0.448**	----	----	----
	TNF	93	-0.329**	0.329	-0.329**	----	----	----	----	----	----	----	----
	IL-2	92	-0.318*	0.386	-0.241*	----	----	----	----	----	0.230*	----	----
	IL-6	93	-0.091	0.091	-0.091	----	----	----	----	----	----	----	----
	IL-10	93	0.135	0.327	0.109	----	----	----	-0.436**	-0.447**	----	----	----

The levels of each cytokine were set as objective variables, and the following factors were considered candidate explanatory variables: age, sex, number of comorbidities (Com), immunosuppression, Eastern Cooperative Oncology Group Performance Status Scale (ECOG-PS) score, serum albumin, estimated glomerular filtration rate (e-GFR), glycated hemoglobin (HbA1c), and booster vaccination type. The values presented in the table are partial correlation coefficients (r_p_) except for those in the columns for *n* (sample size) and R (multiple regression coefficient). The level of significance of r_p_ is indicated by bold letters for *P*<0.05. Single asterisk denotes *P*<0.05, double asterisk denotes *P*<0.01, and triple asterisk denotes *P*<0.001

Abbreviations: *IFN* Interferon, *TNF* Tumor necrosis factor, *IL* Interleukin

^a^The dummy variable “sex” was coded as male = 0 and female = 1

^b^Immunosuppression included receiving steroids, immunosuppressive agents, chemotherapy, or biological therapy

^c^The dummy variable “booster vaccination type” was coded as BNT162b2 (Pfizer-BioNTech) = 0 and mRNA-1273 (Moderna) = 1

### Correlations between cytokine levels and IgG (S-RBD) level and neutralizing antibody activity

**Fig. 4:**
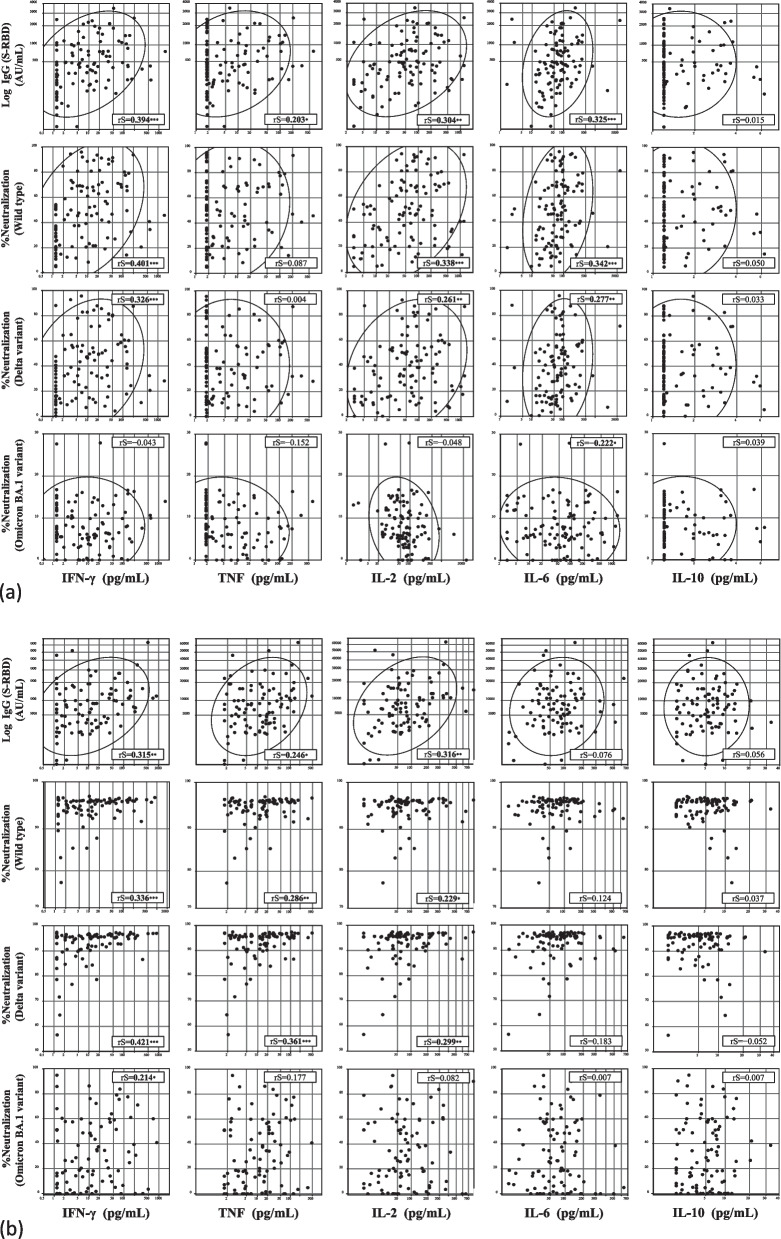
Correlations between cytokine levels and humoral immune responses. Correlations between cytokine levels [interferon (IFN)-γ, tumor necrosis factor (TNF), interleukin (IL)-2, IL-6, and IL-10], serum antibodies to the receptor-binding domain (RBD) of the S1 subunit of the viral spike protein [IgG(S-RBD)] levels, and neutralizing antibody activity of sera against the wild-type virus and Delta and Omicron variants (**a**) six months after primary vaccination and (**b**) three months after booster vaccination. rS: Spearman rank correlation coefficient. Values of rS are shown in bold when P values are less than 0.05: * *P*<0.05, ** *P*<0.01, and *** *P*<0.001. The levels of each cytokine are logarithmically transformed. The 90% confidence ellipse region was drawn assuming a near-Gaussian distribution of the values

Figure 4 shows the correlations of cytokine levels with serum IgG (S-RBD) levels and the neutralizing antibody activity of sera against the wild-type virus and the Delta and Omicron variants. Six months after the primary vaccination, IgG (S-RBD) levels showed a positive correlation with IFN-γ, TNF, IL-2, and IL-6 levels (rS: 0.394, 0.203, 0.304, and 0.325, respectively). Neutralizing antibody activity against the wild-type virus and the Delta variant showed a positive correlation with IFN-γ, IL-2, and IL-6 levels (rS: 0.401, 0.338, and 0.342, respectively, for wild-type; rS: 0.326, 0.261, and 0.277, respectively, for Delta). Neutralizing antibody activity against the Omicron variant showed a negative correlation with IL-6 (rS: -0.222). Three months after the booster vaccination, IgG (S-RBD) levels showed a positive correlation with IFN-γ, TNF, and IL-2 levels (rS: 0.315, 0.246, and 0.316, respectively). Neutralizing antibody activity against the wild-type virus and the Delta variant showed a positive correlation with IFN-γ, TNF, and IL-2 levels (rS: 0.336, 0.286, and 0.229, respectively, for wild-type; rS: 0.421, 0.361, and 0.299, respectively, for Delta). Neutralizing antibody activity against the Omicron variant showed a positive correlation with IFN-γ (rS: 0.214). IL-10 levels exhibited no notable correlation with IgG (S-RBD) levels or neutralizing antibody activity six months after the primary vaccination or three months after the booster vaccination.

### Correlations between IFN-γ, TNF, IL-2, IL-6, and IL-10 levels

**Fig. 5:**
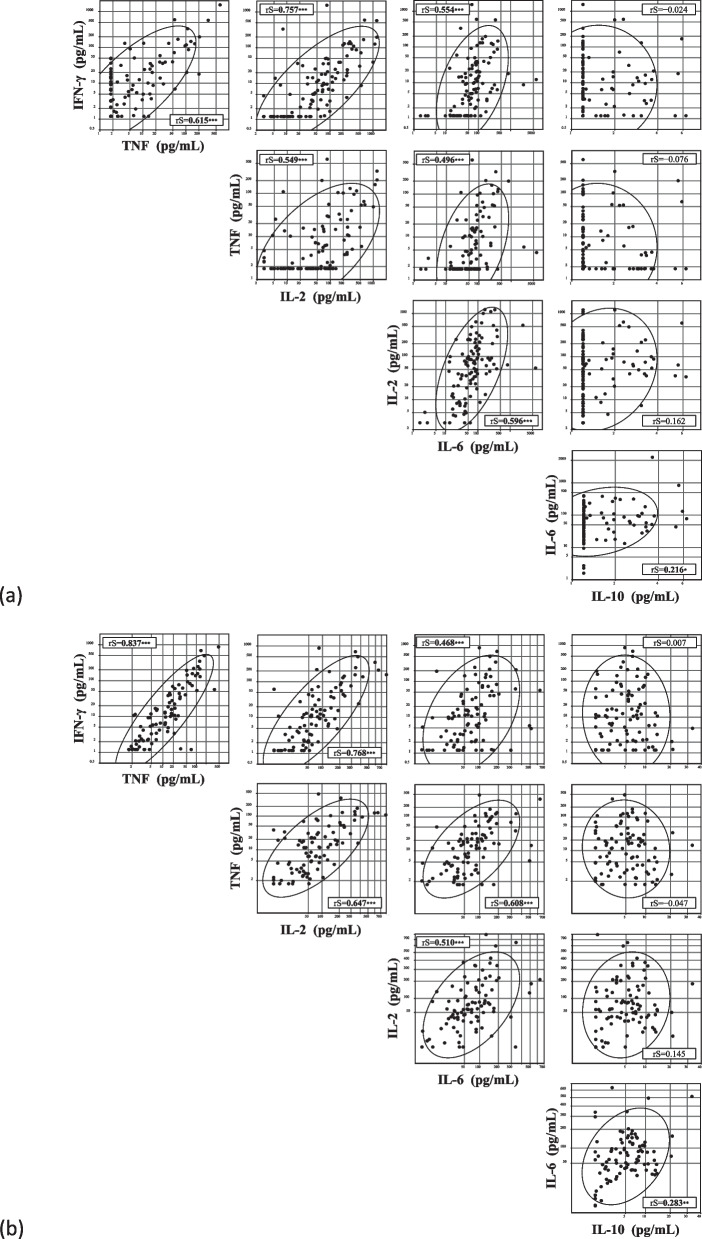
Correlations between interferon (IFN)-γ, tumor necrosis factor (TNF), interleukin (IL)-2, IL-6, and IL-10 levels. Correlations between IFN-γ, TNF, IL-2, IL-6, and IL-10 levels at (**a**) six months after primary vaccination and (**b**) three months after booster vaccination. rS: Spearman rank correlation coefficient. Values of rS are shown in bold when P values are less than 0.05: * *P*<0.05, ** *P*<0.01, and *** *P*<0.001. The levels of each cytokine are logarithmically transformed. The 90% confidence ellipse region was drawn assuming a near-Gaussian distribution of the values

Figure 5 shows pairwise correlations among IFN-γ, TNF, IL-2, IL-6, and IL-10 levels. Six months after the primary vaccination (Fig. 5a), IFN-γ levels were positively correlated with TNF, IL-2, and IL-6 levels (rS: 0.615, 0.757, and 0.554, respectively); TNF levels with IL-2 and IL-6 levels (rS: 0.549 and 0.496, respectively); IL-2 levels with IL-6 levels (rS = 0.596); and IL-6 levels with IL-10 levels (rS = 0.216). Three months after booster vaccination (Fig. 5b), IFN-γ levels were positively correlated with TNF, IL-2, and IL-6 levels (rS: 0.837, 0.768, and 0.468, respectively); TNF levels with IL-2 and IL-6 levels (rS: 0.647 and 0.608, respectively); IL-2 levels with IL-6 levels (rS = 0.510); and IL-6 levels with IL-10 levels (rS = 0.283). No significant correlation was found between IL-10 levels and IFN-γ, TNF, or IL-2 levels after the primary or booster vaccination.

## Discussion

This prospective longitudinal study, conducted over 48 weeks, aimed to assess the dynamics of pro- and anti-inflammatory spike-specific PBMC responses post COVID-19 mRNA vaccinations among residents of LTCFs. Our study sought to determine if older, severely frail individuals, such as LTCF residents, can develop and sustain cellular immunity over time similar to younger, healthier individuals following COVID-19 vaccination. Additionally, the study aimed to investigate whether vaccination induces an anti-inflammatory cellular immune response. This study is unique in several respects. First, it included LTCF residents with advanced age (median age: 89.0 years, IQR: 83.0-93.0 years) and severe frailty (57.9% had an ECOG-PS score of 4 and 29.8% had an ECOG-PS score of 3). It demonstrated that the cellular immune response of very old and severely frail individuals differs significantly from that of older individuals living independently in the general community. Second, it employed a prospective longitudinal design, tracking the same participants over the span of one year. Finally, this study assessed cellular immune responses not only following the primary vaccination series but also after booster vaccination, marking a novel approach compared to prior reports.

T-cells play a crucial role in controlling SARS-CoV-2 infection, and growing evidence suggests that they may help prevent or limit disease severity [19, 47]. We observed a lower magnitude of pro-inflammatory spike-specific PBMC responses in LTCF residents and outpatients than in healthcare workers following primary vaccination, suggesting that the level of cell-mediated immunity following COVID-19 vaccination in older and more vulnerable individuals may be lower than that in healthy younger populations. These findings align with those of previous studies employing various methodologies such as measurement of cytokine levels in cell culture supernatants, ELISpot or FluoroSpot T-cell assays, and intracellular cytokine staining using flow cytometry. These studies have consistently reported diminished T-cell reactions in older individuals [12, 48–51]. It is important to note that our data represent cytokine levels measured in the supernatants of spike protein peptide-stimulated PBMCs, which may include contributions from multiple cell types and not exclusively T-cells.

Although mortality rates from COVID-19 have decreased to levels comparable to those of influenza in younger individuals, they are still higher in older individuals [2]. This may be because of a weak cellular immune response following COVID-19 vaccination in the older population. The mitigation of SARS-CoV-2 infection control measures is progressing in the general healthy population; however, it may still be necessary to continue infection control measures in LTCFs to prevent outbreaks.

Booster vaccinations may not significantly enhance cell-mediated immunity, as evident from the absence of significant increases in IFN-γ, IL-2, and IL-6 levels in all examined subgroups, except for IL-2 levels in outpatients following booster vaccination, when compared with the levels before the booster vaccination. This contrasts with the results of a previous study conducted with the same participants [13], where booster vaccination elicited a significant increase in neutralizing antibody activity against the wild-type and Delta variants. Notably, although humoral immunity may wane over time, cell-mediated immunity established via COVID-19 vaccination tends to last for an extended period [14–17]. T-cells can confer prolonged immunity to conserved SARS-CoV-2 epitopes, thereby potentially safeguarding against severe illnesses caused by diverse viral variants [14–17]. Thus, in terms of enhancing cellular immunity, the first booster vaccination (the third dose) may have played a limited role. However, we were unable to establish this point because we did not assess data from individuals who did not receive the booster. In the absence of booster vaccination, pro-inflammatory spike-specific PBMC responses could have diminished over time, whereas a booster shot might have helped preserve levels similar to those experienced after the primary vaccination.

On the other hand, booster vaccination may partially improve pro-inflammatory spike-specific PBMC responses in older and more vulnerable individuals to a level comparable to that of the general population. After receiving booster vaccinations, LTCF residents and outpatients exhibited similar IL-2 and IL-6 levels to those of healthcare workers. This confirms previous findings and emphasizes the possible value of repeated vaccinations [52–54]. However, even with booster shots, LTCF residents showed lower levels of IFN-γ and TNF than healthcare workers. Meanwhile, the levels in outpatients were similar to those in healthcare workers. Therefore, a single booster vaccination may not be sufficient to increase cell-mediated immunity in older, severely frail populations. These findings suggest that the ideal booster intervals differ between older severely frail individuals and younger healthy individuals. Older severely frail individuals may require more frequent booster vaccinations than healthy younger individuals. However, the optimal interval to administer booster vaccinations is unknown and requires clarification in future studies.

IL-10 is widely recognized as a key inhibitor of adaptive T-cell responses [55, 56] and exhibits lung-protective activity during bacterial and viral infections [57–63]. Severe COVID-19 shows a distinct inflammatory phenotype with blunted anti-inflammatory responses, including IL-10 levels, in contrast to elevated pro-inflammatory cytokine levels [64]. This implies the crucial role of IL-10 in managing COVID-19 severity by counterbalancing the inflammatory response. In our study, IL-10 levels increased significantly after booster vaccinations but not after the primary series. Booster vaccinations may enhance IL-10 levels, contributing to a balanced immune response, potentially reducing severe inflammatory reactions and complications. Our findings suggest that the benefits of booster vaccinations extend beyond increasing antibody titers; they also enhance regulatory mechanisms controlling hyperinflammation. This dual role in humoral and cellular responses highlights the importance of booster vaccinations in protecting against severe COVID-19 outcomes. Moreover, the IL-10 levels following booster vaccination in LTCF residents did not significantly differ from those observed in healthcare workers or outpatients, indicating that booster vaccinations elicit a comparable anti-inflammatory response in both older, more vulnerable individuals, and in the general population.

One of the leading theories on aging proposes that older organisms often exhibit a pro-inflammatory state, marked by elevated levels of circulating inflammatory biomolecules [65, 66]. Although inflammation is essential for combating infections, persistent and prolonged inflammation can detrimentally affect health. This chronic, non-infectious inflammation associated with aging, known as inflammaging, contributes to a decline in immune function, termed as immunosenescence [65]. The weak post-vaccination immune response in the LTCF residents in this study could be due to inflammaging. However, we did not assess the inflammaging status of the participants, so this remains speculative. This important issue requires further investigation in future studies.

Inflammaging may exacerbate the immune response during SARS-CoV-2 infection, potentially amplifying the cascade that leads to cytokine storms, which could result in tissue damage and severe COVID-19 outcomes.

Recent studies have underscored the long-term benefits of vaccination in decreasing inflammation after breakthrough infections. The research tracked the longitudinal concentrations and trajectories of 21 cytokines and chemokines, including IL-2RA, IL-7, IL-8, IL-15, IL-29 (interferon-λ), inducible protein-10, monocyte chemoattractant protein-1, and TNF-α, and showed that fully vaccinated individuals displayed significantly lower concentrations of these markers than unvaccinated individuals during the onset phase of symptomatic COVID-19 [67]. This evidence suggests that COVID-19 vaccination may blunt the elevation of cytokine and chemokine concentrations and potentially shorten the duration of pro-inflammatory responses following SARS-CoV-2 infection. This could partly explain how COVID-19 vaccination helps to reduce severe morbidity and mortality. Vaccinated individuals may exhibit lower cytokine and chemokine concentrations due to the presence of neutralizing antibodies and antibody avidity maturation, which could reduce viral load and modulate the inflammatory response. Our study further suggests that anti-inflammatory spike-specific cellular immune responses also contribute to mitigating excessive inflammatory responses.

In this study, higher levels of IFN-γ, IL-2, and IL-6 following primary vaccination were associated with increased serum IgG (S-RBD) levels and neutralizing antibody activity against the wild-type virus and Delta variant, suggesting that these pro-inflammatory spike-specific PBMC responses play an important role in eradicating the virus partially by inducing the production of antibodies by B cells. The booster shot may initiate a balanced immune response involving both pro-inflammatory and anti-inflammatory components, enabling the eradication of the virus and avoiding excessive inflammation.

As the immune system ages, it undergoes senescence that may lead to a diminished response to vaccines [68]. These alterations result in impaired immune functions, including limited germinal center responses, reduced numbers of naive cells, amplified memory cell populations, and increased inflammatory subsets of adaptive immune cells [69–72]. The present findings show large inter-individual differences in spike-specific PBMC responses following COVID-19 vaccination and imply that age is associated with a decrease in cellular immunity. Age exhibited negative correlations with levels of pro-inflammatory cytokines, including IFN-γ, TNF, IL-2, and IL-6, following COVID-19 vaccination.

However, it remains unclear whether immune senescence is solely related to chronological age or whether other factors also play a role. As shown in the correlation chart in Fig. 3, chronological aging alone cannot fully account for this diversity. To investigate the potential factors responsible for the variability in cytokine levels, we used MRA and found that no significant factors other than chronological age were associated with these individual differences except for TNF levels six months after primary vaccinations and IFN-γ, IL-2, and IL-10 levels three months after booster vaccination. It was difficult to identify the significant factors responsible for variability in spike-specific PBMC responses because of the small sample size in this study. Further studies are required to determine the factors associated with the diversity in cellular immunity.

There are some limitations in this study. First, although this study identified spike-specific PBMC responses as a potential indicator of the cellular immune response, it did not provide a clear understanding of how this cellular response translates to actual immune protection against the development of severe disease. Although the presence of neutralizing antibodies is indicative of protection against infection [73, 74], it is unclear whether the extent of cellular immune responses in vitro is associated with protection against severe diseases. Further research is crucial for developing a better understanding of how cellular immune responses may correlate with immune protection, and future studies may include correlation analyses between cellular responses and tangible disease outcomes. Second, the reason for the difference between humoral and cellular immune responses following booster vaccination is currently unknown. Our previous research demonstrated that booster vaccination significantly attenuated individual differences in neutralizing antibody activity against the wild-type virus and the Delta variant [13]. However, in this study involving the same participants, considerable individual differences in spike-specific PBMC responses persisted even after booster vaccination. The potential for repeated booster vaccinations to diminish these variations in spike-specific PBMC responses awaits further exploration in subsequent studies. Finally, cellular sources of cytokines were not identified in the present study. Regulatory T-cells (Tregs) play important roles in suppressing inflammation and maintaining immune homeostasis. Future research should investigate the CD4+ T-cell subtypes elicited by vaccination, examining if and how the ratio of Th1, Th2, and Tregs varies over time after repeated vaccinations, and if there are any disparities present in different age groups or populations. Answering these questions could provide a more comprehensive understanding of the CD4+ T-cell response to COVID-19 vaccination and potentially lead to the development of more effective vaccines.

## Conclusions

Older and more vulnerable individuals may exhibit inferior cell-mediated immunity following COVID-19 vaccination compared to the general population. A single booster vaccination may not adequately enhance cell-mediated immunity in this demographic. Elevated IL-10 levels post-booster vaccination suggest that the booster may trigger both pro-inflammatory and anti-inflammatory cellular immune responses, potentially regulating harmful hyperinflammation. Our results could be useful for the development of robust booster strategies as protective measures against the development of severe COVID-19 in older and severely frail individuals, such as LTCF residents.

### Supplementary Information

Additional file 1: Baseline characteristics of the study participants. The distribution of demographic characteristics and data on coexisting conditions among participants at baseline are shown in Additional file 1.

Additional file 2: Recruitment of participants, testing, and follow-up. The number of participants included in the final analysis who underwent assessment of spike-specific PBMC responses at each period is shown in Additional file 2. This study included a prospective cohort of long-term care facility (LTCF) residents, outpatients, and healthcare workers. During the study period (March 5, 2021, to July 6, 2022), the participants provided peripheral blood samples for the assessment of spike-specific PBMC responses before the primary vaccination and at 24 and 48 weeks after the primary vaccination. One participant refused to complete both vaccination doses and was thus excluded from the final analysis. The remaining participants completed two vaccination doses with the BNT162b2 (Pfizer-BioNTech) coronavirus disease 2019 (COVID-19) vaccine in the primary vaccine series (two intramuscular doses of 30 mcg, each given three weeks apart). From 24 to 48 weeks after the primary vaccination, two participants failed to receive booster vaccinations and were excluded from the final analysis at 48 weeks. The remaining participants received booster vaccinations from 24 to 48 weeks after the primary vaccination. Therefore, the assessment at 48 weeks after the first dose constituted an assessment approximately three months after the booster vaccination, wherein all healthcare workers, 14 of 26 outpatients, and 15 of 50 LTCF residents received the BNT162b2 (Pfizer-BioNTech) COVID-19 vaccine, and 12 of 26 outpatients and 35 of 50 LTCF residents received the mRNA-1273 (Moderna) COVID-19 vaccine. The vaccine types for booster vaccinations for healthcare workers and LTCF residents were specified by local governments.

## Data Availability

No datasets were generated or analysed during the current study.

## Abbreviations

IFN: Interferon
TNF: Tumor necrosis factor
IL: Interleukin;
IQR: Interquartile range
ECOG-PS: Eastern Cooperative Oncology Group Performance Status Scale
FIM: Functional independence measure
MMSE: Mini-Mental State Examination
WBC: White blood cell count
AST: Aspartate aminotransferase
ALT: Alanine transaminase
e-GFR: estimated glomerular filtration rate
SARS-CoV-2: Severe acute respiratory syndrome coronavirus 2
COVID-19: Coronavirus disease 2019;
Th1: T helper type 1
Th2: T helper type 2
RBD: Receptor-binding domain
S-RBD: RBD of the S1 subunit of the viral spike protein
sVNT: surrogate virus neutralization test
MRA: Multiple regression analysis
rS: Spearman's rank correlation coefficient
LTCF: Long-term care facility
Ig: Immunoglobulin
