# Supplementary material for: Kinetics of pro- and anti-inflammatory spike-specific cellular immune responses in long-term care facility residents after COVID-19 mRNA primary and booster vaccination: a prospective longitudinal study in Japan
Source: Immun Ageing. 2024 Jun 22;21:41. doi: 10.1186/s12979-024-00444-1 (PMC11193299; doi:10.1186/s12979-024-00444-1)
Supplement: Supplementary file 1 — Additional file 1: Baseline characteristics of the study participants. The distribution of demographic characteristics and data on coexisting conditions among participants at baseline are shown in Additional file 1. [file 12979_2024_444_MOESM1_ESM.docx]

**Additional file 1. Baseline characteristics of the study participants**

|  |  |  | Healthcare workers | | Outpatients | | Residents of  long-term care facilities | | P value ^*1^ |
| --- | --- | --- | --- | --- | --- | --- | --- | --- | --- |
|  |  |  | (*n* = 21) | | (*n* = 28) | | (*n* = 57) | |  |
|  |  |  |  |  |  |  |  |  |  |
| **Age, years, (IQR)** | | | 51.0 | (42.0–60.0） | 72.0 | (66.5–76.0) | 89.0 | (83.0–93.0) | <0.001 |
| **Age group, years, *n* (%)** | | |  |  |  |  |  |  |  |
|  | < 45 | | 8 | (38.1) | 0 | (0.0) | 0 | (0.0) | <0.001 |
|  | 45 to < 65 | | 9 | (42.9) | 5 | (17.9) | 2 | (3.5) |  |
|  | 65 to < 85 | | 4 | (19.0) | 22 | (78.6) | 15 | (26.3) |  |
|  | ≥ 85 | | 0 | (0.0) | 1 | (35.7) | 40 | (70.2) |  |
| **Sex, no. (%)** | | |  |  |  |  |  |  |  |
|  | Male | | 7 | (33.3) | 22 | (78.6) | 15 | (26.3) | <0.001 |
|  | Female | | 14 | (66.7) | 6 | (21.4) | 42 | (73.7) |  |
| **Body mass index, (IQR)** | | | 21.8 | (20.9–23.2） | 22.7 | (20.7–23.9) | 18.90 | (17.1–21.1) | <0.001 |
| **ECOG-PS, *n* (%)** | | |  |  |  |  |  |  |  |
|  | 0 |  | 21 | (100.0) | 21 | (75.0) | 0 | (0.0) | <0.001 |
|  | 1 |  | 0 | (0.0) | 7 | (25.0) | 0 | (0.0) |  |
|  | 2 |  | 0 | (0.0) | 0 | (0.0) | 7 | (12.3) |  |
|  | 3 |  | 0 | (0.0) | 0 | (0.0) | 17 | (29.8) |  |
|  | 4 |  | 0 | (0.0) | 0 | (0.0) | 33 | (57.9) |  |
| **Comorbidity, *n* (%)** | | |  |  |  |  |  |  |  |
|  | Chronic respiratory diseases | | 0 | (0.0) | 25 | (89.3) | 9 | (15.8) | <0.001 |
|  | Chronic heart diseases | | 0 | (0.0) | 1 | (3.6) | 17 | (29.8) | <0.001 |
|  | Chronic liver diseases | | 0 | (0.0) | 2 | (7.1) | 2 | (3.5) | 0.522 |
|  | Chronic kidney disease | | 2 | (9.5) | 12 | (42.9) | 26 | (45.6) | 0.007 |
|  | Cerebrovascular diseases | | 1 | (4.8) | 3 | (10.7) | 23 | (40.4) | <0.001 |
|  | Hypertension | | 6 | (28.6) | 13 | (46.4) | 32 | (56.1) | 0.092 |
|  | Diabetes mellitus | | 1 | (4.8) | 8 | (28.6) | 12 | (21.1) | 0.106 |
|  | Solid cancer | | 0 | (0.0) | 4 | (14.3) | 4 | (7.0) | 0.246 |
|  | Leukemia | | 0 | (0.0) | 0 | (0.0) | 0 | (0.0) | NC |
|  | Lymphoma | | 0 | (0.0) | 0 | (0.0) | 2 | (3.5) | 1.000 |
|  | Acquired immunodeficiency syndrome | | 0 | (0.0) | 0 | (0.0) | 0 | (0.0) | NC |
|  | Connective tissue diseases | | 1 | (4.8) | 1 | (3.6) | 0 | (0.0) | 0.211 |
| **Immunosuppression** ^*2^ | | | 1 | (4.8) | 2 | (7.1) | 5 | (8.8) | 1.000 |
| **No. of comorbidities, *n* (%)** | | |  |  |  |  |  |  |  |
|  | 0 | | 13 | (61.9) | 0 | (0.0) | 0 | (0.0) | <0.001 |
|  | 1 | | 5 | (23.8) | 6 | (21.4) | 6 | (10.5) |  |
|  | 2 | | 3 | (14.3) | 10 | (35.7) | 15 | (26.3) |  |
|  | ≥ 3 | | 0 | (0.0) | 12 | (42.9) | 36 | (63.2) |  |
| **Functional independence measure, (IQR)** | | |  |  |  |  |  |  |  |
|  | Motor function | | 91.0 | (91–91） | 91.0 | (91–91) | 17.0 | (13–46) | <0.001 |
|  | Cognitive function | | 35.0 | (35–35） | 35.0 | (35–35） | 15.0 | (8–25) | <0.001 |
|  | Total score | | 126.0 | (126–126） | 126.0 | (126–126) | 32.0 | (21–67) | <0.001 |
| **Mini-Mental State Examination, (IQR)** | | | 30.0 | (30–30） | 27.5 | (26–30) | 7.5 | (0–16) | <0.001 |
| **Laboratory data, (IQR)** | | |  |  |  |  |  |  |  |
|  | Serum total protein level (g/dL) | | 7.5 | (7.2–7.6） | 7.0 | (6.7–7.3) | 6.6 | (6.3–7.2) | <0.001 |
|  | Serum albumin level (g/dL) | | 4.6 | (4.5–4.8） | 4.2 | (4.1–4.3) | 3.6 | (3.4–3.9) | <0.001 |
|  | Serum cholesterol (mg/dL) | | 211.0 | (194.0–234.0） | 195.0 | (169.0–215.5) | 167.0 | (147.0–194.0) | <0.001 |
|  | WBC count/mm^3^ | | 5600 | (4900–7000） | 5650 | (4645–6515) | 5000 | (4000–6540) | 0.178 |
|  | Lymphocyte (%) | | 32.0 | (26.0–36.0） | 28.2 | (24.2–33.3) | 26.7 | (21.1–32.0) | 0.013 |
|  | Hemoglobin (g/dL) | | 14.0 | (12.2–14.6） | 13.4 | (12.8–14.6) | 11.1 | (10.1–12.8) | <0.001 |
|  | Platelet (×10^4^/μL) | | 21.0 | (17.2–24.6） | 21.0 | (15.1–25.3) | 19.8 | (16.3–25.4) | 0.869 |
|  | Total bilirubin (mg/dL) | | 0.6 | (0.5–0.7） | 0.7 | (0.4–0.9) | 0.4 | (0.3–0.5) | <0.001 |
|  | AST (U/L) | | 20.0 | (18.0–27.0） | 22.5 | (19.0–29.0) | 20.0 | (18.0–27.0) | 0.333 |
|  | ALT (U/L) | | 16.0 | (14.0–23.0） | 19.5 | (15.5–31.0) | 14.0 | (10.0–21.0) | 0.004 |
|  | LDH (U/L) | | 168.0 | (144.0–180.0） | 193.0 | (172.5–222.0) | 155.0 | (141.0–183.0) | <0.001 |
|  | e-GFR (mL/min per 1·73 m^2^) | | 78.6 | (68.6–83.7） | 61.9 | (55.0–75.8) | 64.2 | (49.8–92.4) | 0.063 |
|  | HbA1c (%) | | 5.6 | (5.5–5.8） | 6.0 | (5.6–6.3) | 5.4 | (5.2–5.8) | <0.001 |
|  |  |  |  |  |  |  |  |  |  |

The participants in the current study, which examined the cellular immune response, were the same as those in our previous study that investigated the humoral immune response (13). Participants who had undergone at least two assessments of the spike-specific peripheral blood mononuclear cell response from the baseline period were included in the final analysis, resulting in a final sample size smaller than that in our previous study (13).

Abbreviations: IQR, interquartile range; ECOG-PS, Eastern Cooperative Oncology Group Performance Status Scale; WBC, white blood cell; AST, aspartate aminotransferase; ALT, alanine transaminase; LDH, lactate dehydrogenase; e-GFR, estimated glomerular filtration rate; HbA1c, glycated hemoglobin; NC, not calculable

*^1^ Kruskal–Wallis test for continuous variables and Fisher's exact test for categorical variables.

*^2^ Immunosuppression included receiving steroids, immunosuppressive agents, chemotherapy, or biological therapy.
