# Supplementary material for: Kinetics of pro- and anti-inflammatory spike-specific cellular immune responses in long-term care facility residents after COVID-19 mRNA primary and booster vaccination: a prospective longitudinal study in Japan
Source: Immun Ageing. 2024 Jun 22;21:41. doi: 10.1186/s12979-024-00444-1 (PMC11193299; doi:10.1186/s12979-024-00444-1)
Supplement: Supplementary file 2 — Additional file 2: Recruitment of participants, testing, and follow-up. The number of participants included in the final analysis who underwent assessment of spike-specific PBMC responses at each period is shown in Additional file 2. This study included a prospective cohort of long-term care facility (LTCF) residents, outpatients, and healthcare workers. During the study period (March 5, 2021, to July 6, 2022), the participants provided peripheral blood samples for the assessment of spike-specific PBMC responses before the primary vaccination and at 24 and 48 weeks after the primary vaccination. One participant refused to complete both vaccination doses and was thus excluded from the final analysis. The remaining participants completed two vaccination doses with the BNT162b2 (Pfizer-BioNTech) coronavirus disease 2019 (COVID-19) vaccine in the primary vaccine series (two intramuscular doses of 30 mcg, each given three weeks apart). From 24 to 48 weeks after the primary vaccination, two participants failed to receive booster vaccinations and were excluded from the final analysis at 48 weeks. The remaining participants received booster vaccinations from 24 to 48 weeks after the primary vaccination. Therefore, the assessment at 48 weeks after the first dose constituted an assessment approximately three months after the booster vaccination, wherein all healthcare workers, 14 of 26 outpatients, and 15 of 50 LTCF residents received the BNT162b2 (Pfizer-BioNTech) COVID-19 vaccine, and 12 of 26 outpatients and 35 of 50 LTCF residents received the mRNA-1273 (Moderna) COVID-19 vaccine. The vaccine types for booster vaccinations for healthcare workers and LTCF residents were specified by local governments. [file 12979_2024_444_MOESM2_ESM.pptx]

## Slide 1
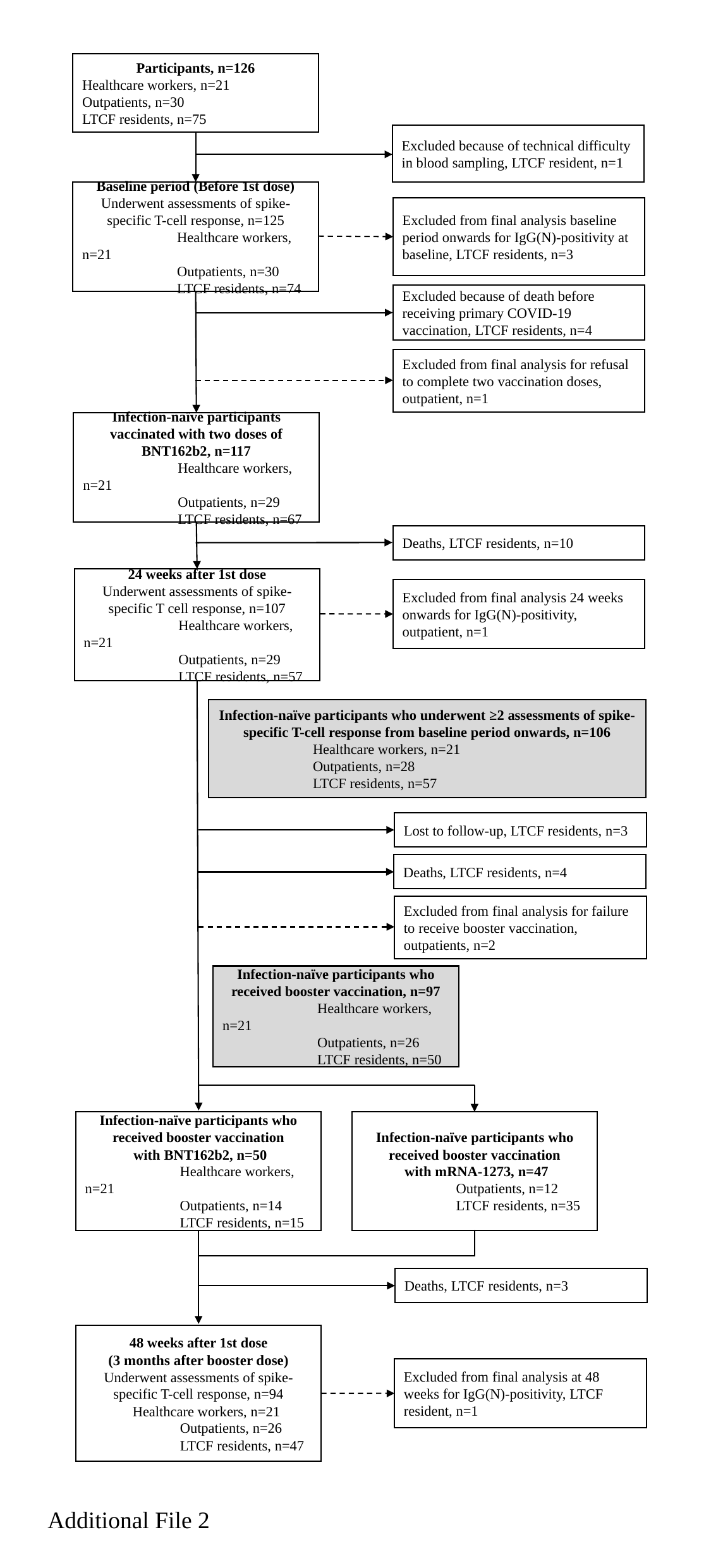

Participants, n=126
Healthcare workers, n=21
Outpatients, n=30
LTCF residents, n=75
Excluded because of technical difficulty in blood sampling, LTCF resident, n=1
Baseline period (Before 1st dose)
Underwent assessments of spike-specific T-cell response, n=125
	Healthcare workers, n=21
	Outpatients, n=30
	LTCF residents, n=74
Excluded from final analysis baseline period onwards for IgG(N)-positivity at baseline, LTCF residents, n=3
Excluded because of death before receiving primary COVID-19 vaccination, LTCF residents, n=4
Excluded from final analysis for refusal to complete two vaccination doses, outpatient, n=1
Infection-naïve participants vaccinated with two doses of BNT162b2, n=117
	Healthcare workers, n=21
 	Outpatients, n=29
	LTCF residents, n=67
Deaths, LTCF residents, n=10
24 weeks after 1st dose
Underwent assessments of spike-specific T cell response, n=107
	Healthcare workers, n=21
	Outpatients, n=29
	LTCF residents, n=57
Excluded from final analysis 24 weeks onwards for IgG(N)-positivity, outpatient, n=1
Infection-naïve participants who underwent ≥2 assessments of spike-specific T-cell response from baseline period onwards, n=106
	Healthcare workers, n=21
	Outpatients, n=28
	LTCF residents, n=57
Lost to follow-up, LTCF residents, n=3
Deaths, LTCF residents, n=4
Excluded from final analysis for failure to receive booster vaccination, outpatients, n=2
Infection-naïve participants who received booster vaccination, n=97
	Healthcare workers, n=21
	Outpatients, n=26
 	LTCF residents, n=50
Infection-naïve participants who received booster vaccination
 with mRNA-1273, n=47
	Outpatients, n=12
 	LTCF residents, n=35
Infection-naïve participants who received booster vaccination
 with BNT162b2, n=50
	Healthcare workers, n=21
	Outpatients, n=14
	LTCF residents, n=15
Deaths, LTCF residents, n=3
48 weeks after 1st dose
(3 months after booster dose)
Underwent assessments of spike-specific T-cell response, n=94
Healthcare workers, n=21
	Outpatients, n=26
	LTCF residents, n=47
Excluded from final analysis at 48 weeks for IgG(N)-positivity, LTCF resident, n=1
Additional File 2
